# Immobilization of β-Galactosidase by Encapsulation of Enzyme-Conjugated Polymer Nanoparticles Inside Hydrogel Microparticles

**DOI:** 10.3389/fbioe.2021.818053

**Published:** 2022-01-13

**Authors:** Narmin Suvarli, Lukas Wenger, Christophe Serra, Iris Perner-Nochta, Jürgen Hubbuch, Michael Wörner

**Affiliations:** ^1^ Biomoleular Separation Engineering, Institute of Process Engineering in Life Sciences, Department of Chemical and Process Engineering, Karlsruhe Institute of Technology (KIT), Karlsruhe, Germany; ^2^ Chimie Macromoléculaire de Précision, Institute Charles Sadron, Université de Strasbourg, Strasbourg, France

**Keywords:** PEG-diacrylate, photopolymerization, affinity binding, enzyme activity, microfluidics, biofunctionalization

## Abstract

Increasing the shelf life of enzymes and making them reusable is a prominent topic in biotechnology. The encapsulation inside hydrogel microparticles (HMPs) can enhance the enzyme’s stability by preserving its native conformation and facilitating continuous biocatalytic processes and enzyme recovery. In this study, we present a method to immobilize β-galactosidase by, first, conjugating the enzyme onto the surface of polymer nanoparticles, and then encapsulating these enzyme-conjugated nanoparticles (ENPs) inside HMPs using microfluidic device paired with UV-LEDs. Polymer nanoparticles act as anchors for enzyme molecules, potentially preventing their leaching through the hydrogel network especially during swelling. The affinity binding (through streptavidin-biotin interaction) was used as an immobilization technique of β-galactosidase on the surface of polymer nanoparticles. The hydrogel microparticles of roughly 400 μm in size (swollen state) containing unbound enzyme and ENPs were produced. The effects of encapsulation and storage in different conditions were evaluated. It was discovered that the encapsulation in acrylamide (AcAm) microparticles caused an almost complete loss of enzymatic activity. Encapsulation in poly(ethylene glycol) (PEG)-diacrylate microparticles, on the other hand, showed a residual activity of 15–25%, presumably due to a protective effect of PEG during polymerization. One of the major factors that affected the enzyme activity was presence of photoinitiator exposed to UV-irradiation. Storage studies were carried out at room temperature, in the fridge and in the freezer throughout 1, 7 and 28 days. The polymer nanoparticles showcased excellent immobilization properties and preserved the activity of the conjugated enzyme at room temperature (115% residual activity after 28 days), while a slight decrease was observed for the unbound enzyme (94% after 28 days). Similar trends were observed for encapsulated ENPs and unbound enzyme. Nevertheless, storage at −26°C resulted in an almost complete loss of enzymatic activity for all samples.

## 1 Introduction

Catalytic properties of enzymes are applied in many fields of modern science and industry: chemical synthesis (Schmid et al., 2001), pharmaceuticals (Vellard, 2003), food (Kuraishi et al., 2001), feed (Lei and Stahl, 2001), detergent (Bisgaard-Frantzen et al., 1999), textile (Doshi and Shelke, 2001) industries, and many more (Hemalatha et al., 2013; Chapman et al., 2018). β-D-Galactosidase (or lactase) is an enzyme present in the human intestine that catalyzes the hydrolysis of lactose and breaks it down into glucose and galactose (Shukla and Wierzbicki, 1975). However, a large part of the global adult population lacks adequate levels of β-galactosidase for the digestion of milk-based products. β-Galactosidase extracted from microbial sources is one of the most relevant enzymes in the food industry and is used in the production of dairy products like ice cream and cheese or for whey hydrolysis (Nguyen et al., 2019).

The widespread applications of enzymes call for the prolongation of their shelf life, reusability, and structural stability (Datta et al., 2013), and the use of enzymes on an industrial scale requires optimizations of their properties. Introduced decades ago, the immobilization of enzymes is still a prominent topic in the worlds of science and industry; various new immobilization methods are published every year (Sastre et al., 2020). The immobilization is a physical or chemical confinement of the enzyme in an environment that can allow its reuse (Brena and Batista-Viera, 2006). In addition, immobilization facilitates the removal of the enzyme from the product, thereby avoiding product contamination. The choice of the immobilization method should always consider the effect on the structure of the enzyme and its native biological function (Selvarajan et al., 2019). The immobilization of enzymes can be achieved via adsorption, affinity binding, covalent attachment, chemical aggregation, entrapment, or encapsulation (Selvarajan et al., 2019). Affinity binding is an immobilization technique based on physical interactions with an excellent selectivity and minimal changes of the enzyme’s conformation (Mohamad et al., 2015). This type of binding provides high retention of the enzyme activity (Roy and Gupta, 2006). Hydrogels and hydrogel beads (Betigeri and Neau, 2002; Lee et al., 2008; Park et al., 2015) have already demonstrated their suitability for the immobilization of enzymes. Nanoparticles are also widely used to immobilize enzymes (Johnson et al., 2008; Cipolatti et al., 2014).

Immobilization of β-D-galactosidase via adsorption is a simple method that supposedly involves ionic interactions, thereby making the effectivity of the immobilization and the enzyme activity yield susceptible to small changes in the nature of the enzyme or the buffer (Ureta et al., 2021). Entrapment immobilization of β-D-galactosidase tends to improve pH and temperature stability, but the supports used for this immobilization technique cannot be reused when the enzyme activity is lost (Souza et al., 2019). Many commercially available supports have been introduced over the years for covalent binding immobilization. On one hand, this method is one of the most studied immobilization techniques for β-D-galactosidase; on the other hand, it usually requires an additional treatment of the support with a reactive compound (Nguyen et al., 2019).

Nanomaterials are suitable carriers for enzyme immobilization due to their chemical stability, high loading density (due to their large surface area), low minimal mass transfer resistance, etc. (Kim et al., 2006; Cipolatti et al., 2014). Immobilization on magnetic nanoparticles may improve the activity of the enzyme and its tolerance to pH, temperature and substrate concentration (Kouassi et al., 2005). Nanoparticles based on gold (Villalonga et al., 2005) and titanium oxide (Bang et al., 2011) among other systems have shown substantial improvement in catalytic properties in the process of bioenzymatic nanoimmobilization. Polymer nanomaterials, such as chitosan nanoparticles (Klein et al., 2012) and poly(methyl methacrylate) (Valério et al., 2015) nanoparticles have many benefits as support materials for enzyme immobilization: easy synthesis methods (usually, one single reaction) and high colloidal stability in suspension.

Aerosol photopolymerization is an easy, eco-efficient and continuous method for the synthesis of highly pure and dry polymer nanoparticles that does not require the use of hazardous organic solvents, surfactants, or heating (Akgün et al., 2013). In other methods, e.g., emulsion polymerization, additional separation procedures are required to obtain pure nanoparticles for enzyme conjugation. The combination of aerosol photopolymerization with thiol-ene monomers ensures the synthesis of spherical cross-linked polymer nanoparticles with reactive -SH groups (Suvarli et al., 2021b) that are not present on other types of nanoparticles such as silicon or gold nanoparticles. A two-step bioconjugation process that can bind various biomolecules onto -SH groups of polymer nanoparticles via thiol-ene “click” reactions was previously introduced (Suvarli et al., 2021a).

Immobilizing enzymes via entrapment in hydrogel microparticles is another method to preserve enzymatic activity for long periods of time. Poly(ethylene glycol) (PEG)-based hydrogels have found many biotechnological applications due to their high water content, hydrophilicity, and biocompatibility (West and Hubbell, 1995). Enzymes encapsulated inside hydrogel microparticles made from interpenetrating polymer networks of PEG and poly(acrylamide) have not shown a significant loss of activity (Lee et al., 2008). Some other hydrogels have even shown enhancement of the activity of encapsulated enzyme compared to the unbound enzyme (Zhang et al., 2016).

Microfluidic devices are state-of-the-art systems that (in combination with curing) can be used to produce polymer particles from monodisperse emulsions (Serra et al., 2007). Among other benefits, avoiding the use of surfactants and easy control of the particle size are fundamental advantages for the application of the device in this study. Particles of different shapes, sizes and compositions can be produced by changing the design and parameters of the microfluidic system (Serra and Chang, 2008). Previous research has already succeeded in encapsulating gold and silver nanoparticles inside polymer microparticles using microfluidic devices (Yu et al., 2019). Dang et al. have successfully encapsulated polystyrene-based microbeads inside monodisperse hydrogel microparticles using a flow-focusing microfluidic device paired with a UV-irradiation source (Dang et al., 2012).

In this study, the activity of β-galactosidase immobilized by bioconjugation on the surface of polymer nanoparticles and encapsulated inside hydrogel microparticles was investigated. The combination of immobilization methods (conjugated on nanoparticles and encapsulated in hydrogel) aims at identifying synergistic effects. Nanoimmobilization preserves the enzyme’s activity for a long time; encapsulation in hydrogel microparticles preserves the enzyme’s native conformation and offers reusability.

Polymer nanoparticles were synthesized via aerosol thiol-ene photopolymerization (Suvarli et al., 2021a). The accessible -SH groups on the surface of these polymer nanoparticles offer an effective way of bioconjugation through a thiol-ene “click” reaction with maleimide-biotin and subsequent addition of streptavidin derivatives (Suvarli et al., 2021a; Suvarli et al., 2021b). An affinity binding immobilization technique was applied with biotin-conjugated nanoparticles and a commercially available streptavidin-β-galactosidase. The conjugated nanoparticles were then introduced into a hydrogel precursor solution. This suspension was used as a dispersed phase in microfluidic emulsion photopolymerization to produce hydrogel microparticles with enzyme-conjugated nanoparticles inside. The impact of the encapsulation in different hydrogels on enzymatic activity was studied and the impact of hydrogel components in enzymatic inactivation was evaluated. Storage studies at different temperatures were performed to investigate the long-term stability of the produced microparticles compared to free unbound enzyme and enzyme-conjugated polymer nanoparticles.

## 2 Materials and Methods

### 2.1 Chemicals

Trimethylolpropane tris (3-mercaptopropionate) (**Trithiol**, Sigma-Aldrich, 95%) and trimethylpropane triacrylate (**TMPTA**, Sigma-Aldrich, contains 600 ppm monomethyl ether hydroquinone as inhibitor) were used as thiol and alkene monomers in aerosol photopolymerization. 2-Methyl-4′-(methylthio)-2 morpholinopropiophenone (**MT-2MP**, Sigma-Aldrich, 98%) was used as a photoinitiator in the aerosol photopolymerization process. Biotin-Maleimide (**b-M**, ≥ 95% (TLC) powder, Merck KGaA) and streptavidin-β-galactosidase (**E**, 150 units/mg, ThermoFisher Scientific) were used for bioconjugation reactions. β-Galactosidase from *Aspergillus oryzae* (β**-gal**, ≥ 8 units/mg solid, Sigma-Aldrich), 2-nitrophenol (**ONP**, Sigma-Aldrich) and 2-nitrophenyl β-D-galactopyranoside (**ONPG**, ≥ 98%, enzymatic, Sigma-Aldrich) were used for enzyme activity assays. Poly(ethylene glycol)-diacrylate (**PEG-DA**, average Mn 575, Sigma-Aldrich), acrylamide (**AcAm**, ≥ 99%, Sigma-Aldrich), N, N′-methylene bis(acrylamide) (**BisAc**, 99%, Sigma-Aldrich) and lithium-phenyl-2,4,6-trimethylbenzoylphosphinate (**LAP**, ≥ 95%, Sigma-Aldrich) were used in the dispersed phase of the microfluidic device to produce hydrogel microparticles. Silicone oil (viscosity 500 cst at 25°C, Sigma-Aldrich) was used as a continuous phase. Poly(ethylene glycol) M_n_ 600 (**PEG**, for synthesis, Merck) was used as a non-reactive alternative of PEG-DA for exposure studies. Phosphate buffer (100 mM, pH 7) was prepared using 38 mM Na_2_HPO_4_ and 68 mM NaH_2_PO_4_.

### 2.2 Experimental Methods

#### 2.2.1 Synthesis of Polymer Nanoparticles

Polymer nanoparticles (50–1,000 nm) were synthesized via aerosol thiol-ene photopolymerization. Trithiol and TMPTA monomers were utilized in 1:1 stoichiometry of functional groups (3*SH:3*C=C) in order to achieve spherical and individual nanoparticles. 1.15 g of Trithiol and 0.85 g of TMPTA were combined in a spray solution flask, 40 g of ethanol (EtOH) was added to the flask and stirred, followed by 0.02 g of MT-2MP photoinitiator. The spray solution flask was then placed inside the aerosol generator (TOPAS^®^, ATM220) which was connected to the photoreactor consisting of two UV-fluorescent devices (T-15.C, Vilber Lourmat, λ_max_ = 312 nm) facing each other and a tube reactor located in between the UV-light sources. The mean residence time of the aerosol inside the reactor was 28 s. One 2 h reaction resulted in almost 100 mg of polymer nanoparticle powder collected on 0.1 μm pore size Durapore^©^ hydrophobic membrane filters.

#### 2.2.2 Bioconjugation of Polymer Nanoparticles

**FIGURE 1:**
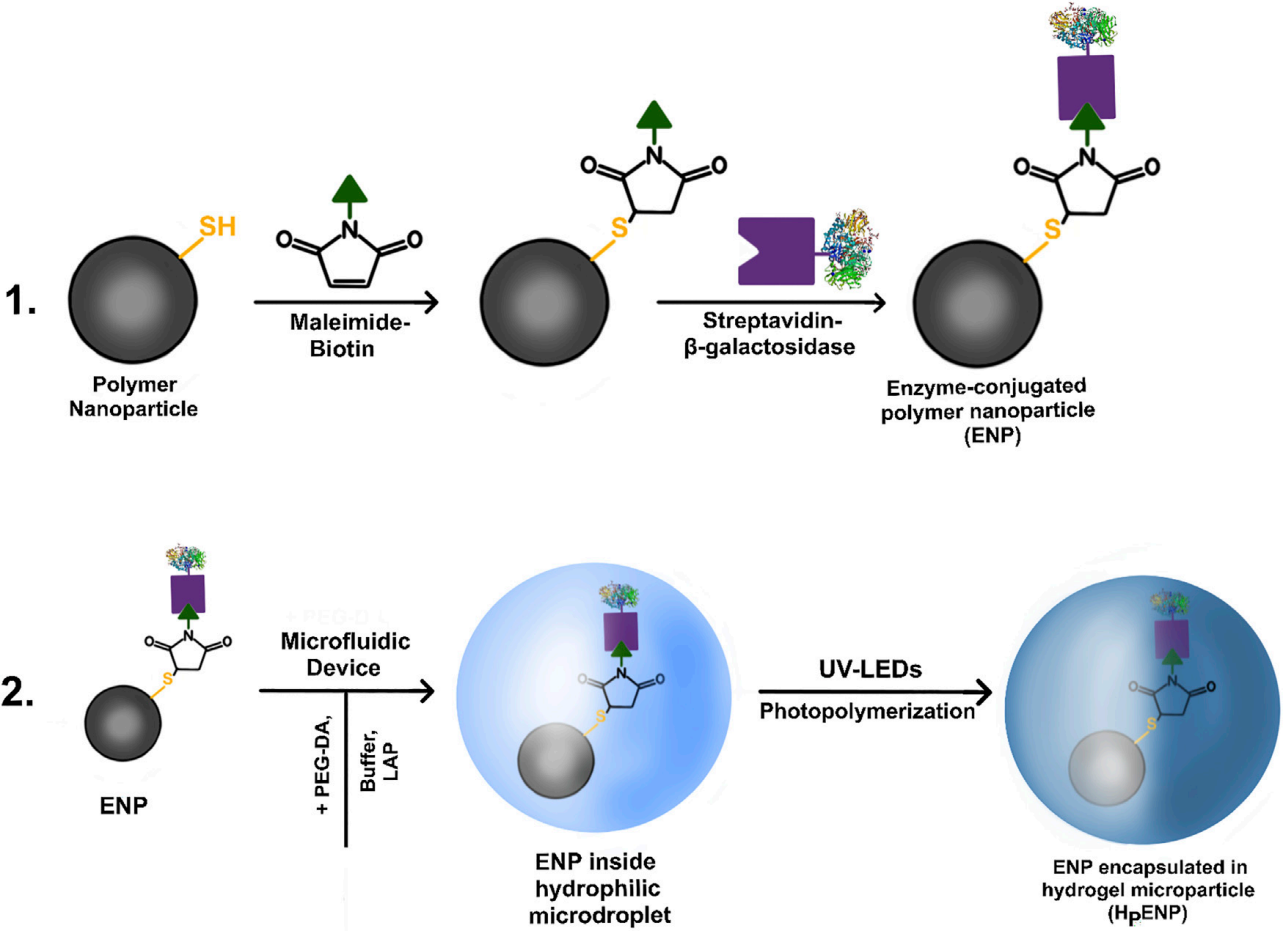
A diagram of **(1)** immobilization of enzyme via two-step bioconjugation process, **(2)** encapsulation of enzyme-conjugated nanoparticles inside hydrogel microparticles using microfluidic device and UV-LEDs.

The polymer nanoparticles produced via aerosol thiol-ene photopolymerization were tested for the presence of reactive -SH groups with Ellman’s reaction. After the test confirmed the presence of -SH groups, the polymer nanoparticles were introduced into a two-step bioconjugation reaction: first, conjugation of biotin-maleimide (biotinylation) via thiol-Michael addition reaction, and second, conjugation of streptavidin-β-galactosidase to biotin (Figure 1).

For the bioconjugation with biotin-maleimide (b-M), 10 mg of dry polymer nanoparticles were dispersed in 9 ml of buffer (3 vol.% of dimethylsulfoxide (DMSO) in 100 mM phosphate buffer, pH 7.0, from here on, referred to as a buffer). 5 mg of b-M was diluted in 1 ml of buffer and added dropwise to the dispersion of nanoparticles under vigorous stirring. The reaction was carried out for 20 h. The produced dispersion was centrifuged at 15,000 g for 10 min and the residual particles were purified with 3 × 10 ml of buffer to remove the unreacted biotin-maleimide. The purified nanoparticles were redispersed in buffer resulting in 10 ml of a 1 mg/ml dispersion for the next bioconjugation step.

The second step of the bioconjugation was carried out with 50 units of streptavidin-β-galactosidase diluted in 1 ml of buffer. The solution was added dropwise to the dispersion of b-M-conjugated polymer nanoparticles under mild stirring. The reaction was carried out for 28 h. The resulting ENPs mixture was purified via centrifugation at 10,000 g for 10 min with 3 × 10 ml of buffer. The enzymatic activity of the supernatants of each purification step and the purified nanoparticles were tested via enzymatic activity assays using ONPG as substrate.

To assess the necessity of the two-step bioconjugation and to exclude the possibility of unspecific binding of the enzyme to the nanoparticles, a control experiment was performed with β-galactosidase instead of streptavidin-β-galactosidase (E). The nanoparticles (10 mg) were dispersed in 4.5 ml of buffer and 0.5 ml of enzyme solution (15 units/ml) was added to the dispersion. The mixture was incubated for 28 h under the same conditions as the second step of the two-step conjugation. The purification of E_control_NPs was also carried out following the purification protocol of ENPs.

#### 2.2.3 Synthesis of Hydrogel Microparticles via Microfluidic Device

**FIGURE 2:**
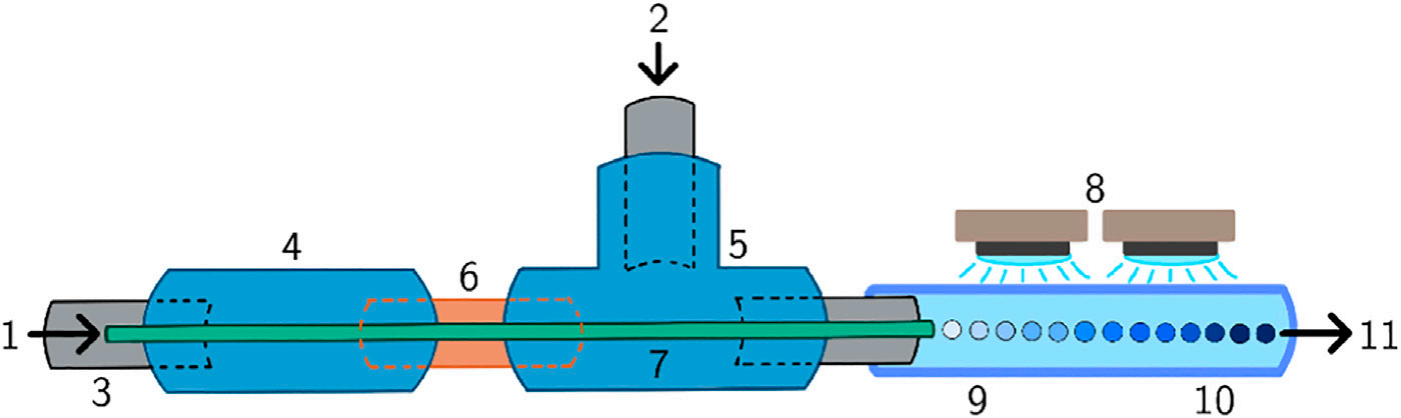
Schematic representation of the microfluidic device. **(1)** Dispersed phase inlet, **(2)** continuous phase inlet, **(3)** PTFE tube, **(4)** connection unit, **(5)** T-junction, **(6)** capillary sleeve, **(7)** capillary, **(8)** UV LEDs, **(9)** microdroplets, **(10)** microparticles, **(11)** outlet.

An axisymmetric needle/tubing microfluidic device (Serra et al., 2007) with a set of UV-LED spots, as pictured in Figure 2, was employed to produce microdroplets and polymerize them resulting in hydrogel microparticles. Dispersed phase (1) was introduced through a micro-scale diameter capillary (7), and the continuous (2) phase was introduced perpendicularly. The microfluidic device consisted of a set of polytetrafluorethylene (PTFE) tubes (0.75 mm inner diameter (ID), Upchurch Scientific) (3), and perfluoralkoxy-alkane (PFA) tubes (1.59 mm ID, Upchurch Scientific), a PEEK connection unit (0.020 inch ID, Upchurch Scientific) (4), a PEEK T-junction (0.040 inch ID, Upchurch Scientific) (5), a flexible fused silica capillary (150 μm ID, 363 μm outer diameter (OD), TSP standard polyimide coating, Molex^®^) (7) and a capillary sleeve (for 340–380 μm capillaries, Upchurch Scientific) (6). Microdroplets were formed on the tip of the capillary and irradiated with an intensity of 17.5 mW/cm^2^ by a set of four UV-LEDs (single color 365 nm LZ4 emitter, I_f,max_ = 1,000 mA, LED Engin) connected in series (8). The irradiation initiated the photopolymerization reaction of the microdroplets (9) resulting in polymer microparticles (10). The dispersed phase was loaded into a 5 ml syringe which was placed inside a Nemesys 290N syringe pump (Cetoni GmbH). The silicone oil was loaded into a 20 ml syringe which was placed inside the syringe pump, as well. The flow rate of the hydrogel precursor solution was 0.05 ml/min, and the flow rate of the silicone oil was 1 ml/min. Under these settings, the microdroplets/microparticles were exposed to UV light for roughly 5 s.

#### 2.2.4 Encapsulation of Enzyme and Enzyme-Conjugated Nanoparticles Inside Hydrogel Microparticles

To ensure the production of comparable samples of free unbound enzyme (E) and enzyme-conjugated nanoparticles (ENPs), the activities of the purified ENPs suspension and the E stock solution were determined. Based on the results, the concentrations of ENPs and E for the production of hydrogel microparticles were chosen to result in an equivalent final activity. The final concentration of 1 mg/ml ENPs corresponded to a free unbound enzyme concentration of 3.11 units/ml.

For ENPs encapsulated in PEG-DA hydrogel microparticles (H_P_ENPs) and AcAm hydrogel microparticles (H_A_ENPs) the concentrations of enzyme were chosen to be the same as for the unbound enzyme encapsulated inside PEG-DA hydrogel microparticles (H_P_E) and AcAm hydrogel microparticles (H_A_E).

For H_P_ENPs, 0.446 ml of dispersion of 5 mg/ml of enzyme-conjugated polymer nanoparticles in buffer was mixed with 2 g of PEG-DA, 2.5 g of buffer and 5 mg of photoinitiator (LAP). The final concentration of ENPs inside the hydrogel microparticles was roughly 1 mg/ml. Encapsulation of unbound enzyme into PEG-DA hydrogel microparticles (H_P_E) was carried out with the same quantities of PEG-DA, buffer and LAP and 0.446 ml of buffer containing enzyme. The final concentration of E inside the hydrogel microparticles was roughly 3.11 units/ml (Figure 1).

For H_A_ENPs, 0.446 ml of dispersion of 5 mg/ml of enzyme-conjugated polymer nanoparticles in buffer was mixed with 2 g of AcAm, 0.2 g of BisAc, 2.5 g of buffer and 5 mg of LAP. Encapsulation of unbound enzyme into AcAm hydrogel microparticles (H_A_E) was carried out with the same quantities of AcAm, BisAc, buffer and LAP and 0.446 ml of buffer containing enzyme. The resulting final concentrations of ENPs and E inside the AcAm microparticles were identical to the PEG-DA microparticles.

To purify the HMPs, the microparticle dispersion (in silicone oil) was centrifuged at 5,000 rpm for 5 min and the silicone oil was physically removed. The residual oil was washed out with 20 ml of xylene three times. The remaining xylene was evaporated on air (for a short time to avoid drying of the hydrogel) and the HMPs were washed three times in buffer. The microparticles were then redispersed in buffer at a ratio of 1:1 by volume. It is important to note that the microparticles were already slightly swollen (from washing cycles) when they were redispersed in buffer.

### 2.3 Analysis Methods

#### 2.3.1 Light Microscopy

An inverted light and fluorescence microscope (Zeiss Axio Observer Z1, Carl Zeiss Microscopy GmbH) with EC Epiplan-Neofluar 2.5X M27 objective was used for imaging of the synthesized hydrogel microparticles in order to define their size and observe the success of encapsulation of ENPs inside the hydrogel microparticles. The swollen microparticles (purified with xylene and dispersed in buffer) were placed on microscope slides and the images were taken using the software ZEN blue (Version 3.3, Carl Zeiss Microscopy GmbH).

#### 2.3.2 Scanning Electron Microscopy

The polymer nanoparticles made by synthesis via aerosol photopolymerization were analyzed with a LEO1530 scanning electron microscope (Carl Zeiss Microscopy GmbH). The dry nanoparticles were dispersed in acetone and distributed on silicon wafers which were sputtered with platinum. The images were taken at a working distance of 5.8 mm and an acceleration voltage of 5 kV.

#### 2.3.3 Enzyme Activity Studies

The comparison studies were carried out with the following samples containing both free and immobilized enzyme:

• free unbound enzyme—streptavidin-β-galactosidase (**E**)
• free enzyme-conjugated polymer nanoparticles (**ENPs**)
• enzyme-conjugated polymer nanoparticles encapsulated in PEG-based hydrogel microparticles (**H**_**P**_ **ENPs**)
• enzyme-conjugated polymer nanoparticles encapsulated in AcAm-based hydrogel microparticles (**H**_**A**_ **ENPs**)
• unbound enzyme encapsulated in PEG-based hydrogel microparticles (**H**_**P**_ **E**)
• unbound enzyme encapsulated in AcAm-based hydrogel microparticles (**H**_**A**_ **E**)

Ten sets of microplates (96 well UV-Star^®^, Greiner Bio-One GmbH) were prepared with equal volumes of these samples (in triplicates with 40 μl of buffer solution/dispersion per well). Samples with either buffer, AcAm-based or PEG-based microparticles (without enzyme or ENPs, 1:1 volume concentration in buffer) were prepared in the same set of plates as the enzyme-containing samples to allow the generation of ONP calibration curves. All plates were sealed with adhesive aluminum foil (Axygen^®^ PCR-AS-200) for storage. One plate was analyzed immediately after the preparation of the microparticles to establish a reference allowing the determination of relative activities. The remaining samples were stored at three different temperatures (−26°C, 8°C and 22°C) for three different durations (1, 7 and 28 days) to analyze the change in enzymatic activity during storage. After the designated storage time, 100 μl of different concentrations of ONP (0, 1, 2.5, 5, 7.5 and 10 mM) were added to the calibration samples and their absorbance at 460 nm was measured using a Tecan^©^ Infinite M200 plate reader. The measurements of the required calibration data were followed by the analysis of the enzymatic activity. To determine the enzymatic activity, 100 μl of 20 mM ONPG as a substrate were added to the enzyme-containing samples, and the absorbance at 460 nm was recorded at 25°C for 60 min.

**FIGURE 3:**
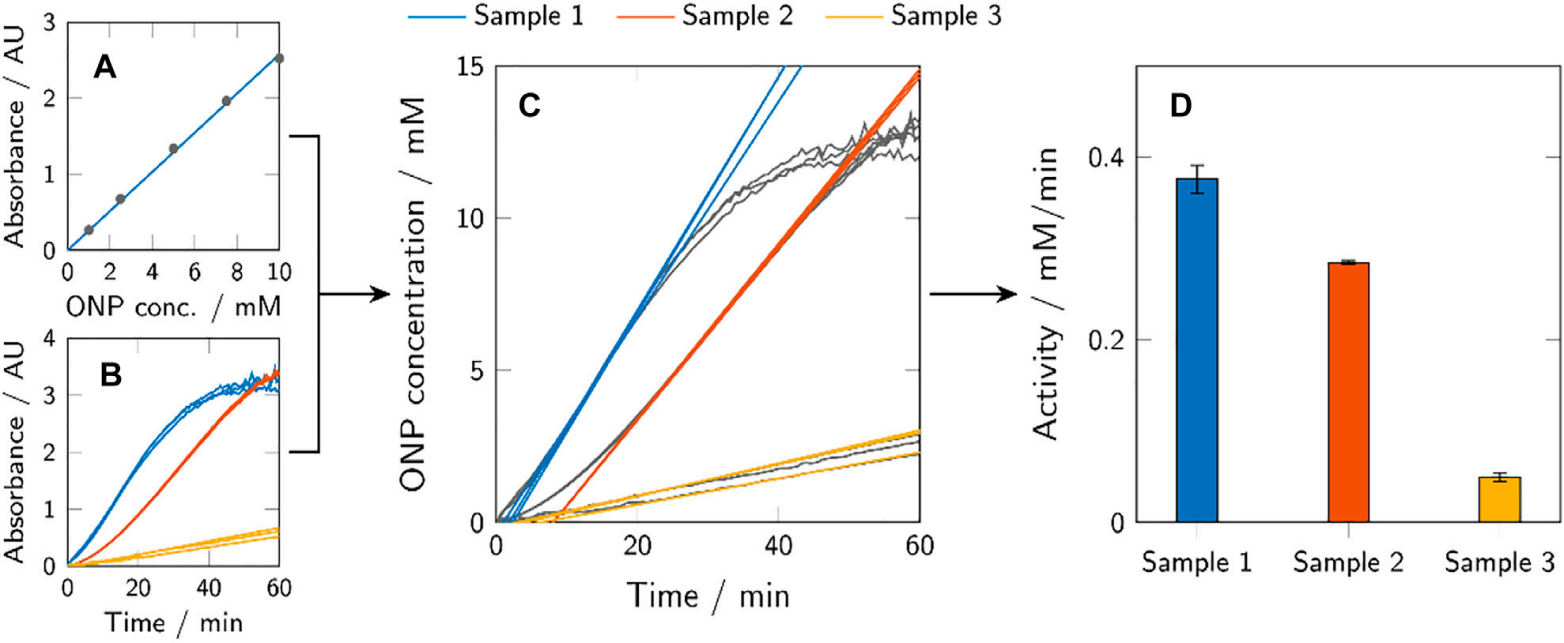
Graphic representation of the activity assay evaluation method. ONP calibration curves were calculated from known ONP samples **(A)** to transform the obtained absorbance data of the activity assays **(B)** to ONP concentration profiles over time **(C)**. The maximum activity of a sample (*a*_max_) corresponds to the maximum slope of the respective curve and was determined using a 12 min rolling-window analysis. The obtained values of the maximum activity were presented using bar plots **(D)**. Depicted here are generic samples.

The results of the activity assays were evaluated using MATLAB R2020a (The MathWorks, Inc.). ONP calibration curves were generated from the absorbance data of the samples with known ONP concentration, as exemplarily shown in Figure 3A. Based on these curves, the ONP concentrations of the activity assays could be calculated. Examples of the absorbance data of three different samples (in triplicates) are shown in Figure 3B and the calculated ONP concentrations in Figure 3C. The resulting graphs of ONP concentration over time often showed an initially reduced slope, before reaching a slope maximum after a delay of several minutes. The maximum slope of a curve corresponds to the maximum enzymatic activity (*a*_max_) and was determined for each sample individually by fitting the curve with a linear fit in a 12 min rolling-window analysis. The determined fits of maximum activity are indicated in Figure 3C, as well. The determined maximum activity can be depicted in common bar plots (Figure 3D).

### 2.4 Exposure Studies

Exposure studies were carried out to identify the mechanisms responsible for the reduction of β-galactosidase activity observed after the photopolymerization process. Specifically, the effect of acrylamide, PEG, the photoinitiator LAP and UV light (and combinations of several factors and components) on the activity of β-galactosidase were evaluated. PEG was chosen to mimic the effect of PEG-DA without the ability to polymerize. A series of samples with the same amount of β-gal and different combinations of PEG, AcAm and LAP was prepared. The applied concentrations of the components were chosen to be representative of the microparticle production process (0.2 g/ml of PEG, 1 M of AcAm, 0.01 wt. % of LAP relative to PEG or AcAm concentration). One set of samples was additionally exposed to UV light for roughly 5 s as in the microparticle production process, a second set was not exposed to UV as a control. Activity studies with all samples were carried out following the same protocol as stated in section 2.3.3.

## 3 Results and Discussion

### 3.1 Conjugation of Enzymes on the Surface of Polymer Nanoparticles

**FIGURE 4:**
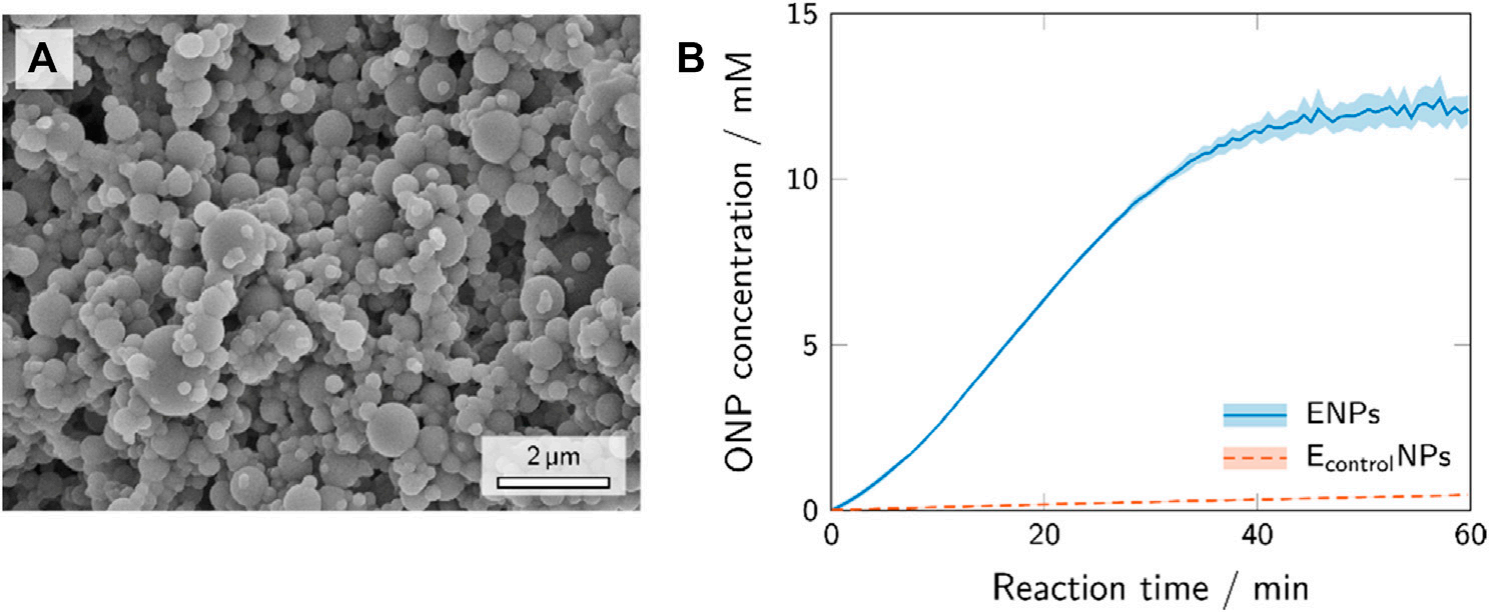
SEM image of the polymer nanoparticles after the synthesis via aerosol photopolymerization **(A)** and the activity assays of the enzyme-conjugated polymer nanoparticles and polymer nanoparticles with unspecifically adhered enzyme, i.e., control experiment **(B)**.

The SEM image (Figure 4A) provides information on the shape (spherical, no heavy agglomeration) and size (30–1,000 nm) of the polymer nanoparticles synthesized from TMPTA and Trithiol via aerosol thiol-ene photopolymerization. These polymer nanoparticles possess reactive -SH groups [confirmed via Ellman’s test (Riddles et al., 1979)] which offer an effective way of bioconjugation with maleimide (Hoyle et al., 2004).

Depending on the method of immobilization, different enzyme-conjugates can be designed to couple with the support (Walt and Agayn, 1994). Immobilization via affinity binding is based on physical interactions. In this paper, application of affinity binding with a streptavidin-β-galactosidase conjugate and polymer nanoparticles conjugated with biotin-maleimide was adopted, because the streptavidin-biotin binding is one of the strongest known non-covalent interactions (Roy and Gupta, 2006). Conjugation of maleimide to polymer nanoparticles synthesized employing aerosol thiol-ene photopolymerization has already been proven effective in previous studies (Suvarli et al., 2021a). In this study, we use a two-step bioconjugation procedure to attach streptavidin-β-galactosidase to the surface of polymer nanoparticles using a thiol-ene “click” reaction (first step, biotinylation) and biotin-streptavidin binding (second step). The nanoparticles conjugated with streptavidin-β-galactosidase showed activity corresponding to 3.11 units/mg of nanoparticles. This implies that approximately 31.1 units conjugated on the surface of the nanoparticles out of 50 units of enzyme introduced into the reaction with 10 mg of nanoparticles (section 2.2.2). The removal of the unreacted enzyme proved to be complete after washing cycles. The supernatants of the washed enzyme-conjugated nanoparticles were tested for presence of enzyme with ONPG, and the washing cycles continued until the amount of enzyme in the supernatant was negligible.

A control experiment mimicking the conjugation reaction with unmodified β-galactosidase instead of streptavidin-β-galactosidase was carried out to confirm that the binding occurs mostly due to specific biotin-streptavidin interaction and not due to unspecific adhesion. The products of the biotin-streptavidin reaction (ENPs) and the control experiment (E_control_NPs) were assayed for their enzymatic activity. The results of these experiments are shown in Figure 4B. The activity assay with the ENPs sample showed a sharp increase in ONP concentration and a sigmoidal curve progression $\left(a_{max}=38.3\pm 0.4\cdot 10^{-2}\;\frac{mM}{min}\right)$ , while the E_control_NPs sample only showed a very low and linear increase in ONP $\left(a_{max}=8.7\pm 0.3\cdot 10^{-3}\;\frac{mM}{min}\right)$ . These results reveal that only a negligible amount of enzyme is unspecifically adhered on the surface of the polymer nanoparticles, compared to a high enzyme conjugation yield via affinity binding. This evinced that a controlled two-step conjugation is an effective tool to specifically conjugate streptavidin-β-galactosidase on the surface of nanoparticles and hardly any unspecific binding of β-galactosidase takes place.

### 3.2 Encapsulation of Enzyme and Enzyme-Conjugated Nanoparticles Inside the Hydrogel Microparticles

The produced ENPs were encapsulated inside hydrogel microparticles using a microfluidic device. A 1 mg/ml dispersion of ENPs in hydrogel precursor solution (dispersed phase) was prepared and injected into a stream of silicone oil (continuous phase) through a capillary in a microfluidic device (Figure 1). The immiscibility of the dispersed and continuous phase leads to the formation of microdroplets of uniform size which polymerize under UV irradiation and form hydrogel microparticles.

One of the key elements of this process was to make the hydrogel precursor dispersions stable so that the polymer nanoparticles do not form aggregates and sediment in the syringe or clog the PEEK capillary. The formulation of the hydrogel precursor dispersion was therefore adjusted (addition of 3 vol.% of DMSO), and the dispersion showed stability for over 4 h.

#### 3.2.1 Microscopic Analysis of Hydrogel Microparticles

**FIGURE 5:**
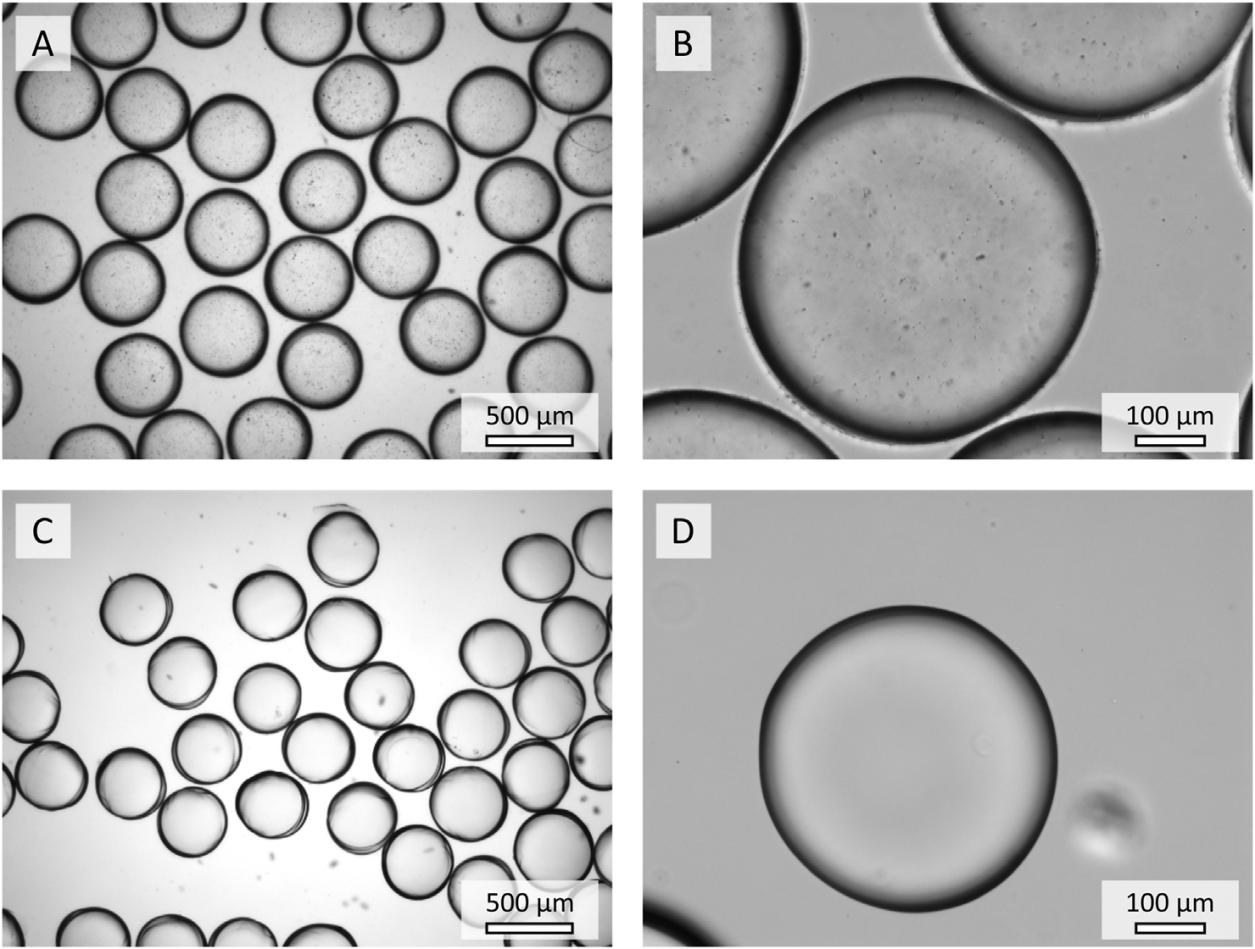
Microscopic images of the hydrogel microparticles containing ENPs **(A,B)** and unbound enzyme **(C,D)**.

Light microscopy images (Figure 5) of PEG-based hydrogel microparticles with enzyme-conjugated nanoparticles (H_P_ENPs, Figures 5A,B) reveal a successful encapsulation of the nanoparticles in contrast to hydrogel microparticles with unbound enzyme (H_P_E, Figures 5C,D). In addition, the nanoparticles inside the H_P_ENPs are well distributed and no large aggregates of nanoparticles are observed. The hydrogel microparticles in the figures are swollen (2 days of swelling in buffer). The sizes of swollen H_P_ENPs and H_P_E show no significant difference ( 403±17 µm and 424±23 µm , respectively). According to previous studies, viscosities of the dispersed and continuous phases affect the size of the microparticles produced in the microfluidic process (Yu et al., 2019), and increasing the viscosity of the dispersed phase will lead to bigger microparticles. The addition of nanoparticles to the hydrogel precursor solution could potentially cause a change in viscosity leading to a shift in microparticle size. The lack of a significant observable size difference between H_P_ENPs and H_P_E indicates that no drastic change in viscosity occured upon the addition of the nanoparticles. Due to the negligible difference in microparticle size in our studies, its effect on enzyme activity was disregarded in the scope of this paper. However, the AcAm-based hydrogel microparticles were bigger (approximately 100 μm difference) than the PEG-based hydrogel microparticles.

#### 3.2.2 Effect of the Encapsulation Process on the Enzymatic Activity

**FIGURE 6:**
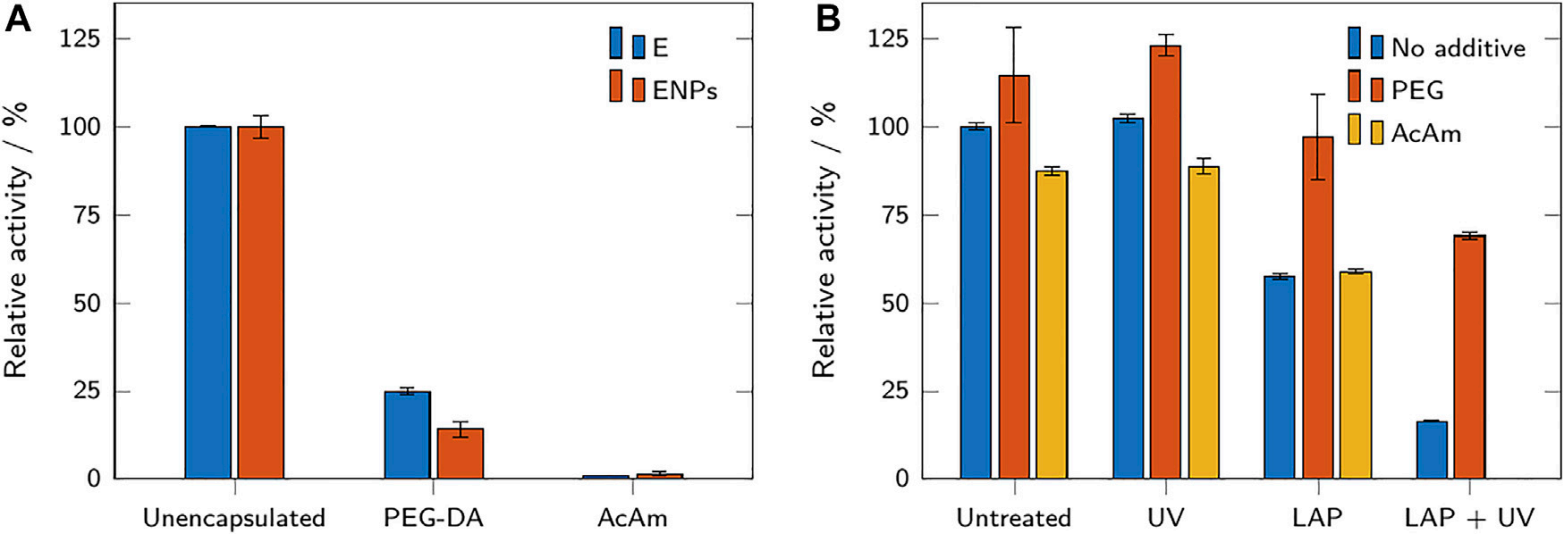
**(A)** Relative activity of enzymes and ENPs in buffer, compared to PEG-DA and acrylamide-based microparticles containing enzyme and ENPs. The relative activities refer to the respective free (not encapsulated) samples. **(B)** Relative activity of free enzyme with and without additives after exposure to the photoinitiator LAP and UV light. The relative activities refer to the untreated sample without additive. All results are shown as mean values ± standard deviation (*n* = 3).

Two types of hydrogel microparticles were prepared: from acrylamide (with bisAc as cross-linker) and poly(ethylene glycol) diacrylate. ENPs and unbound enzyme were encapsulated into these hydrogel microparticles (sample names H_A_ENPs, H_A_E, H_P_ENPs, H_P_E). The activity assays of these microparticles before storage compared to non-encapsulated enzyme and ENPs are presented in Figure 6A. Enzyme and ENPs encapsulated into acrylamide microparticles (H_A_MPs) showed hardly any activity $\left(a_{max}<0.5\cdot 10^{-2}\;\frac{mM}{min}\right)$ , while the H_P_MPs revealed high enzymatic activity with both ENPs $\left(a_{max}=4.6\pm 0.7\cdot 10^{-2}\;\frac{mM}{min}\right)$ and unbound enzyme $\left(a_{max}=7.1\pm 0.3\cdot 10^{-2}\;\frac{mM}{min}\right)$ . Compared to the samples of free unbound enzyme and ENPs, the activities of encapsulated samples exhibit a decrease of roughly 75–85%. A certain reduction in activity is expected due to the mass transfer limitation caused by the HMPs, slowing down the supply of the enzyme with substrate and the removal of product. To counteract this phenomenon, it is desirable to produce smaller particles due to their more favorable surface-area-to-volume ratio. This could be achieved by modifying the flow rates or the capillary size in the microfluidic production process or by using a continuous phase with lower viscosity. Due to the almost identical size of the microparticles produced, the particle size does not explain the observed difference in activity between H_P_MPs and H_A_MPs. Other factors might be the density of the hydrogel polymer networks and the inactivation of the enzyme through interactions with components of the hydrogel precursor solutions, as examined in the following section.

The observed relative activity of H_P_E was higher than the activity of H_P_ENPs (Figure 6A), although the enzyme concentration was adjusted to result in the same activity for both microparticle samples. Leaching of the unbound enzyme may be a potential explanation for the observation of higher activity in H_P_E. Due to its small size, the unbound enzyme may be able to diffuse out of the hydrogel microparticles and exert higher activity in solution due to the reduced mass transfer limitations (Wenger et al., 2020). Although a highly probable explanation to the observed activity change, the study of leaching was not in the scope of this paper.

The ENPs would retain inside the hydrogel network due to their large size. Encapsulated inside the hydrogel microparticles, ENPs might also be covalently bound to the hydrogel network. The conjugation of enzyme of the surface of the nanoparticles does not exclude the possibility of a small concentration of remaining -SH groups on the surface and these -SH groups can, therefore, participate in thiol-ene reactions with PEG-DA during the encapsulation process.

#### 3.2.3 Effect of the Hydrogel Precursor Solutions on the Enzymatic Activity

To examine a possible link between components of the hydrogel precursor solutions and a reduction in enzymatic activity, unmodified β-galactosidase was dissolved in either buffer, a PEG 600 solution, or an AcAm solution. PEG 600 was chosen as a non-polymerizable mimicry of PEG-DA 575. The mixtures were either exposed to UV light, to the photoinitiator LAP, or both simultaneously. Untreated samples served as a control. After exposure, all samples were tested for their enzymatic activity. The results are shown in Figure 6B. The untreated samples show that the addition of PEG was accompanied by a small increase in activity (+15%), while the addition of acrylamide caused a small decrease (−13%). Exposing the samples to UV light did not change the observed activity compared to the untreated sample. The addition of the photoinitiator LAP caused a decrease of activity in all samples, but this decrease was far less pronounced for the PEG sample (−15%) than for the AcAm sample (−33%) or the sample without additive (−43%). Exposure to both UV and LAP caused the strongest reduction in activity (−84% for the sample without additive, −40% for the PEG sample). Due to polymerization, the AcAm sample could not be assayed after exposure to LAP and UV. The results show that the addition of the photoinitiator LAP had a detrimental effect on the residual enzymatic activity of the samples, especially when accompanied by UV exposure. This indicates that free radicals generated during the polymerization process are a major cause of activity loss in the produced HMPs. Reduction of the activity in presence of only LAP is observed because the initiator radicals can also be generated under daylight. The inactivation of enzymes by free radicals has been reported before (Dumitru and Nechifor, 1994). The unchanged activity of the samples exposed to only UV shows that the inactivation is not caused by irradiation and/or heat generated from the UV-LEDs.

The presence of PEG in the sample seems to preserve and enhance the activity of β-galactosidase; the reduction in activity after exposure to LAP and UV is considerably reduced compared to the samples with AcAm or without any additive. Indeed, PEG has been reported to have a stabilizing effect on proteins under certain conditions (Wang, 1999). This may explain the significantly higher activity of H_P_MPs compared to the activity of H_A_MPs and the reduction in enzymatic activity upon encapsulation which was higher for H_P_ENPs than for H_P_E (depicted in Figure 6A). As the stabilizing effect of PEG depends both on its chain length and on protein size, the conjugation of the enzyme to nanoparticles might affect the PEG-enzyme interactions, resulting in a lower “protection” from free radicals.

### 3.3 Storage Studies

**FIGURE 7:**
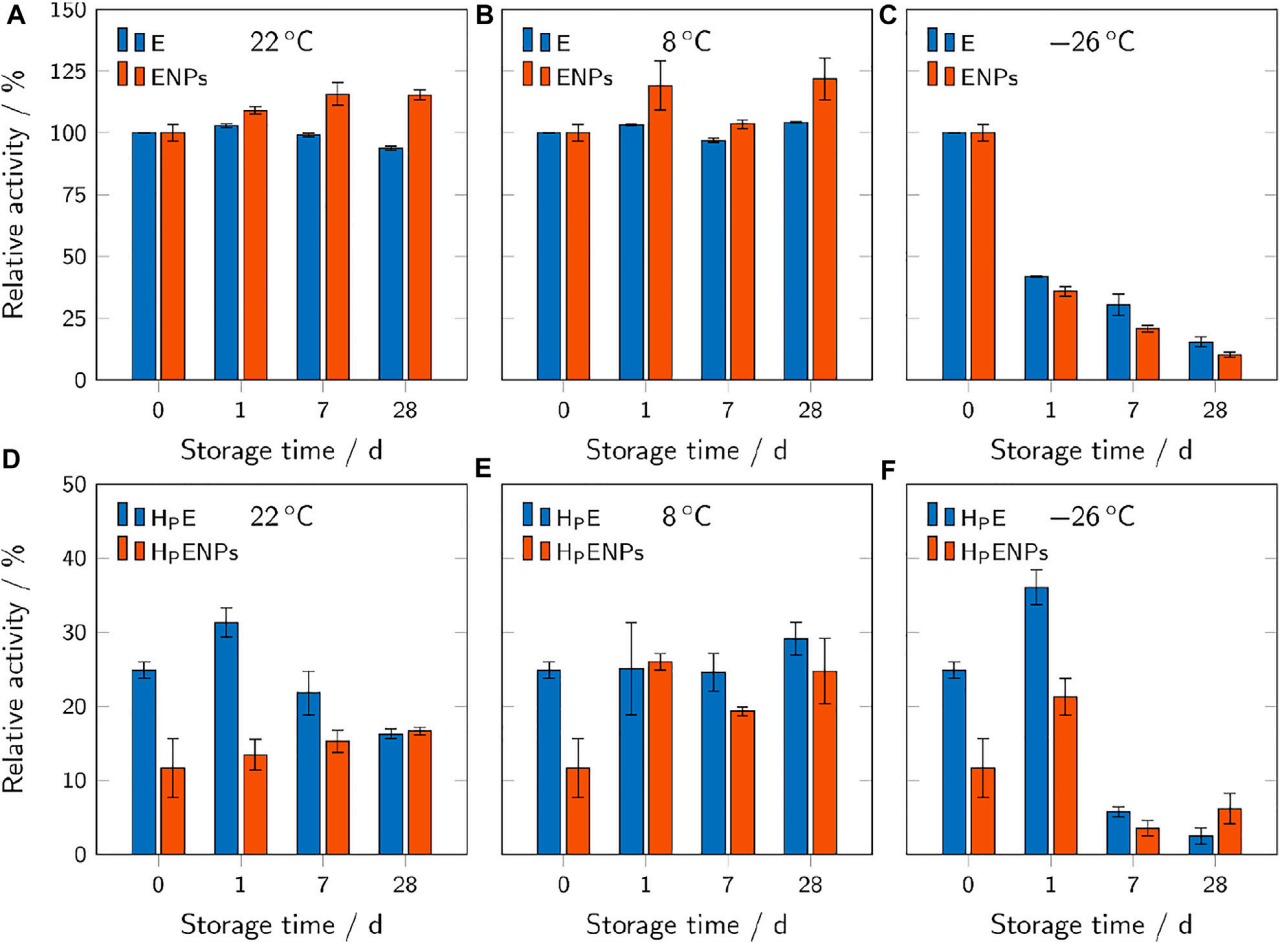
Relative activities of free ENPs and free unbound enzyme **(A–C)**, compared to hydrogel microparticles containing ENPs (H_P_ENPs) and free enzyme (H_P_E) **(D–F).** The samples were stored at different temperatures and for different durations. The relative activities refer to the respective unencapsulated samples before storage and are shown as mean values ± standard deviation (*n* = 3).

Storage studies were carried out to evaluate the stability and reactivity of HMPs and ENPs at different storage temperatures and over time. Samples were stored in sealed microplates at different temperatures (22, 8 and −26°C) for 1, 7 and 28 days and the residual activity was determined at different time points. Figure 7 shows the results for free unbound enzyme and ENPs (A-C) compared to unbound enzyme and ENPs encapsulated in PEG-based HMPs (D-F). The results of AcAm-based HMPs are not shown due to their low residual activity even before storage.

#### 3.3.1 Storage at Room Temperature

At 22°C, free unbound enzyme showed a slight downward trend of activity over storage time with a residual activity of 94% after 28 days (Figure 7A). Free ENPs showed the opposite trend, even increasing their activity to 115% after 28 days. This indicates that the immobilization of enzyme on the surface of polymer nanoparticles may be beneficial regarding the retention of enzymatic activity over time at room temperature. However, the only minimal decrease in activity of the free unbound enzyme shows relatively high stability of streptavidin-β-galactosidase at room temperature.

The same trends (decreasing activity over time for E, increasing activity for ENPs) were observed for samples of unbound enzyme and ENPs encapsulated inside the hydrogel microparticles (Figure 7D). Although the encapsulation of unbound enzyme and ENPs inside the hydrogels leads to a significant decrease in activity, the microparticles still provide reusability which must be considered when assessing the overall effect of the encapsulation process. The loss in activity of the H_P_E over time cannot be explained without additional extensive research on this topic. Leaching of the enzyme during swelling may counteract the loss of the activity to a certain degree because the leached unbound enzyme is not subjected to mass transfer limitations and can exert a higher activity. This may explain the spike in activity on day 1.

No concrete statements can be made about the cause of the observed increase of enzyme activity in ENPs over time. Improved enzymatic activity in an immobilized form (compared to the unbound form) has already been observed with some other enzymes. Lipase immobilized via adsorption and in presence of detergents showed increased activity compared to the native enzyme. This phenomenon was attributed to the different (open and closed) conformations of lipase in immobilized and native forms (Mateo et al., 2007). In the case of β-galactosidase, a conformational change due to immobilization, if any, would have appeared before storage (at storage time 0, Figures 7A–C). The activity increase in nanoimmobilized β-galactosidase might be due to conformational changes induced by buffer-nanoparticle interactions in ENPs dispersions over time.

#### 3.3.2 Storage in the Fridge

Unlike at 22°C, the free unbound enzyme samples showed no decrease in activity at 8°C (Figure 7B). The free ENPs showed the same increasing trend as at 22°C.

The behavior of unbound enzyme in H_P_E is also relatively unchanged throughout 28 days, whereas the activity of H_P_ENPs shows a more than two-fold increase after 1 day (Figure 7E). The difference between the results of storage of H_P_E at room temperature and at 8°C can be due to the lower degree of swelling of hydrogels, studied previously by Urushizaki et al. (Fumio et al., 1990) on poly(vinyl alcohol) based hydrogels at lower temperatures (5°C). If swelling is dependent on temperature in PEG-based hydrogels, further studies can be carried out to investigate the release of enzyme from the hydrogel at various temperatures.

#### 3.3.3 Storage in the Freezer

Samples stored at −26°C showed a significant decrease in enzymatic activity over time (Figures 7C,F), making this storage condition unsuitable for the analyzed samples. The activity of the free unbound enzyme and free enzyme-conjugated nanoparticles (Figure 7C) is reduced by more than 50% after 1 day and by more than 75% after 28 days. Several factors may contribute to the massive loss of activity upon freezing. Low temperatures are able to cause the denaturation of enzymes even without freezing (Hatley and Franks, 1989). Also, the formation of ice crystals may result in a severe shift in pH (from 7.0 to 3.8 in a 100 mM phosphate buffer solution) and thereby cause the inactivation of β-galactosidase (Pikal-Cleland et al., 2000) which has limited stability in the acidic pH range (Ustok et al., 2010).

The results of storage of H_P_E and H_P_ENPs in the freezer are more inconsistent. After 1 day in the freezer, the microparticles show an enhancement in activity, whereas longer storage leads to a significant drop of activity. Extremely low temperatures could also affect the hydrogel’s structure and swelling properties, thereby affecting the enzyme conformation and activity (Morelle et al., 2018).

### 3.4 Further Remarks

The presented study demonstrates the feasibility of producing hydrogel microparticles with embedded enzyme-conjugated nanoparticles and indicates the potential benefits and limitations of the method. Future studies should address the identified challenges in several areas.

The obtained results show that the encapsulation within hydrogel microparticles caused a significant loss in enzymatic activity. While this can potentially be compensated by improved reusability, it is still desirable to preserve maximum activity by optimizing the hydrogel content and photopolymerization process which have been identified as major contributors to activity loss. Further studies should address the possibility of a reduction in LAP concentration and UV exposure to reduce enzymatic inactivation through free radicals to a minimum. Other parameters to address are the type of photoinitiator and the PEG-DA chain length which may influence both the observed protective effect during polymerization and the cross-linking density of the resulting polymer network. An increase in mesh size of the hydrogel can enhance activity by reducing mass transfer limitations (Jang et al., 2010). This can also be achieved by producing smaller particles with a more favorable surface-area-to-volume ratio. Due to the enhanced production time for smaller particles, a trade-off is required between production throughput and optimization of the produced particles. Optimizing the production process can shift the balance in this trade-off. The microfluidic process could be improved and scaled up by optimizing flow rates and employing parallelized processes. The immobilization of β-galactosidase on the nanoparticles could be switched to a single-step procedure by using different binding chemistry.

The performed storage studies indicated a slight superiority of ENPs over free enzymes regarding storage stability, mainly at room temperature. Future studies should consider the influence of nanoimmobilization on enzyme activity, to address observations of the activity increase after 28 days. Storage studies at elevated temperatures and at more adverse buffer conditions like extreme pH values or high organic solvent content should be carried out to determine the full potential of hydrogel microparticles. Especially in buffers containing organic solvents, encapsulation in hydrogels has been shown to be beneficial for enzyme stability (Maier et al., 2018). For industrial processes, knowledge about the kinetics of the free and immobilized β-galactosidase is essential. Kinetic parameters can be evaluated using integrated reaction rate equations (Goldstein, 1976; Carrara and Rubiolo, 1996). In a previous study, we have already investigated the kinetics of β-galactosidase immobilized in 3D-printed composite hydrogels based on high internal phase emulsions (Wenger et al., 2020).

A major aspect of encapsulating ENPs instead of the unbound enzyme inside hydrogel microparticles was not specifically addressed in the present study: the avoidance of leaching. Enzymes tend to leach from hydrogels over time depending on the size of the enzyme and the mesh size of the hydrogel network (Jang et al., 2010). Attaching the enzyme to a nanoparticle sterically anchors the enzyme within the hydrogel and allows the use of hydrogels with a larger mesh size which reduces mass transfer limitations (Jang et al., 2010). Future studies could investigate the correlations between nanoparticle and hydrogel mesh size and the resulting leaching behavior and activity.

The present study only investigated the conversion of the model substrate ONPG. One of the main “real-world” applications of β-galactosidase in food industry is the production of lactose-free milk (Lartillot, 1993; Haider and Husain, 2007). Future studies could implement the presented method and employ H_P_ENPs in biocatalytic packed-bed reactors for the hydrolysis of lactose present in whey and milk. As an alternative approach, ENPs could be immobilized in 3D-printed, hydrogel-based bioreactors, as has already been demonstrated for β-galactosidase (Radtke et al., 2018; Wenger et al., 2020) and other enzymes (Maier et al., 2018; Peng et al., 2019).

## 4 Conclusion

β-galactosidase was conjugated onto polymer nanoparticles and subsequently encapsulated inside two types of hydrogel microparticles. Polymer nanoparticles were produced via aerosol thiol-ene photopolymerization and the reactive -SH groups on the surface of the nanoparticles were used for functionalization with biotin-maleimide. Streptavidin-β-galactosidase was then conjugated onto the biotin unit via affinity binding immobilization method. The enzyme-conjugated nanoparticles were encapsulated inside hydrogel microparticles using a microfluidic device coupled with UV-LEDs. The size of the produced microparticles was 400–500 μm in diameter after swelling, depending on the nature of the hydrogel (PEG-DA or AcAm).

Our results demonstrate that the encapsulation within AcAm hydrogels resulted in particles with no relevant residual activity, whereas the PEG-DA microparticles preserved a residual activity of 15–25%, compared to the activity of the free unbound enzyme. The reduction in activity could mostly be attributed to enzymatic inactivation during the photopolymerization process which occurred due to the formation of free radicals from the photoinitiator exposed to UV-LEDs. The research highlighted in this paper contributed to the general understanding of activity behavior of β-galactosidase when exposed to a radical photopolymerization reaction for encapsulation. It is shown that UV light (and the heat from the UV lamp) does not significantly affect the activity of β-galactosidase in buffer solution. However, more detailed studies of effects caused by radicals on enzyme activity can improve the encapsulation process. Storage studies show a slight decline in activity over time for free unbound enzyme (94% after 28 days) at room temperature, while the activity of nanoimmobilized enzyme increased to 115%. All tested samples were stable at 8°C and lost most of their activity when stored in a frozen state at −26°C, probably due to a pH shift induced by the employed phosphate buffer and other relevant factors. Encapsulating enzyme-conjugated nanoparticles inside hydrogel microparticles can enable the reusability, however, an improvement of the encapsulation technique is necessary to address the loss of enzymatic activity. Future studies should also address options to reduce the size of the hydrogel microparticles which might reduce mass-transfer limitations. Another focus is the systematic investigation of leaching of enzymes and ENPs and stability studies in harsher conditions, e.g., at elevated temperatures and in organic solvents.

## Data Availability

The original contributions presented in the study are included in the article/supplementary material, further inquiries can be directed to the corresponding author.
